# Screening of MicroRNA Related to Irradiation Response and the Regulation Mechanism of miRNA-96-5p in Rectal Cancer Cells

**DOI:** 10.3389/fonc.2021.699475

**Published:** 2021-08-11

**Authors:** Fengpeng Wu, Bingyue Wu, Xiaoxiao Zhang, Congrong Yang, Chaoxi Zhou, Shuguang Ren, Jun Wang, Yafan Yang, Guiying Wang

**Affiliations:** ^1^Department of Radiation Oncology, Fourth Hospital of Hebei Medical University, Shijiazhuang, China; ^2^Department of Oncology, Hebei Provincial People’s Hospital, Graduate School of Hebei Medical University, Shijiazhuang, China; ^3^Department of General Surgery, Fourth Hospital of Hebei Medical University, Shijiazhuang, China; ^4^Laboratory Animal Center, Fourth Hospital of Hebei Medical University, Shijiazhuang, China; ^5^Department of General Surgery, Third Hospital of Hebei Medical University, Shijiazhuang, China

**Keywords:** rectal cancer, irradiation resistance, miRNA-96-5p, GPC3, Wnt/β-catenin

## Abstract

Neoadjuvant chemoradiotherapy has been widely used in the treatment of locally advanced rectal cancer due to the excellent advantages of irradiation in cancer therapy. Unfortunately, not every patient can benefit from this treatment, therefore, it is of great significance to explore biomarkers that can predict irradiation sensitivity. In this study, we screened microRNAs (miRNAs) which were positively correlated with irradiation resistance and found that miRNA-552 and miRNA-183 families were positively correlated with the irradiation resistance of rectal cancer, and found that high expression of miRNA-96-5p enhanced the irradiation resistance of rectal cancer cells through direct regulation of the GPC3 gene and abnormal activation of the canonical Wnt signal transduction pathway. Based on the radioreactivity results of patient-derived xenograft models, this is the first screening report for radio-resistant biomarkers in rectal cancer. Our results suggest that miRNA-96-5p expression is an important factor affecting the radiation response of colorectal cancer cells.

## Introduction

In the past decade, the application of radiotherapy-based preoperative neoadjuvant chemoradiotherapy (nCRT) has played a major role in improving surgical resection rates and rectal sphincter retention while reducing the rate of local recurrence in patients with locally advanced rectal cancer (LARC). However, only 40-80% of patients can benefit from this treatment, and with such a large variance, no less than 20% of patients will be resistant to nCRT (1–4). In consideration of the foregoing, it is not advantageous to indiscriminately perform nCRT on all LARC patients. Therefore, the screening out of patients with high resistance to radiotherapy before treatment will be conducive to the implementation of individualized precision therapy for LARC patients.

Patient-derived xenograft (PDX) model is a xenograft model constructed by implanting newly excised tumor tissue from patients into immunodeficient mice. Currently, it is widely used in the study of anti-tumor drug screening, but its application in the radiotherapy field has been rarely reported. Previous studies (5–9) have shown that the traditional cell lines used to construct tumor animal models are clonal cell subpopulations with strong proliferation rates cultured in a nutrient rich environment. Animal models constructed by these cells cannot truly reflect the heterogeneity of cancer, nor can it demonstrate a precise reaction of the biological state of tumors under hypoxia and nutrient deprivation. The PDX model not only retains the histological and genetic characteristics of the primary tumor, but also retains the microenvironment of tumor cells, thus overcoming the limitations of the traditional cell lines transplantation model (6, 10, 11). It has even been suggested that tumor stem cells and stem cell-like cells can proliferate in the PDX model (12, 13). Based on the above theory, we constructed the PDX model of rectal cancer and screened the irradiation response ability of rectal cancer tissues from different patients.

MicroRNAs (miRNAs), a family of endogenous short non-coding RNAs, can regulate the translation or induce degradation of specific mRNAs by binding to the 3’-untranslated region of mRNAs (14). Studies have confirmed that the change of their expression not only participates in the occurrence and progression of tumors (15–17), but also regulates the cancer cells responsiveness to irradiation (18–22). To our knowledge, most of the studies on miRNA regulation of irradiation reactivity in LARC patients come from other types of cancers at which the validation of miRNAs relation to radiation reactivity have been confirmed.

In this study, based on the irradiation reactivity analysis in PDX models of LARC, we screened out miRNAs that were positively correlated with irradiation resistance, verified them in different rectal cancer cell lines, and explored the relevant mechanism of miRNA-96-5p enhancing irradiation resistance of rectal cancer cells.

## Materials and Methods

### Characteristics of Patients and Establishment of PDX Model

The study population included 56 LARC patients (39 male and 17 female), with a median age of 57.6 years (41.3-72.7 years), who underwent total mesorectal excision (TME) and refused to accept nCRT in our hospital from July 2015 to July 2016. Postoperative pathology showed that among the 56 patients, 37 had pT3 and 19 had pT4 tumors; in addition to having pT3 and pT4 tumors, 12 patients had pN0, 27 had pN1, and 17 had pN2 diseases. There were 43 patients with R0 resection and 13 with R1 resection. Among them, 29 patients completed more than 50Gy postoperative radiotherapy, and 36 completed more than 6 cycles of chemotherapy with fluorouracil based chemotherapy. As of March 2021, 36 (64.28%) patients have had local recurrence and/or distant metastasis and 31 (55.36%) patients have died.

**Figure 1:**
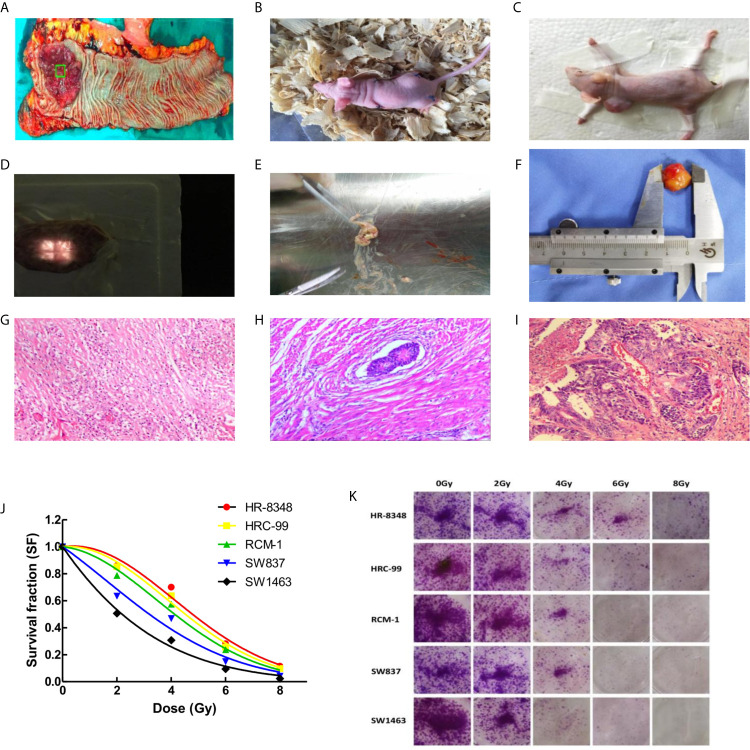
Differential screening of irradiation reactivity to rectal cancer tissues and to rectal cancer cell lines. **(A)** Fresh specimen cut from the center of tumor; **(B)** Rectal cancer tissue was transplanted into nude mice to construct the first generation PDX model; **(C)** Second generation PDX model for screening irradiation reactivity; **(D)** Compensation tissue and irradiation field on the surface of xenograft tumor; **(E)** Irradiation caused liquefaction and necrosis of the tumor; **(F)** Measurement of xenograft tumor *in vitro*; **(G)** Sterilization with marked fibrosis (RCRG1); **(H)** Marked fibrosis but macroscopic disease present (RCRG2); **(I)** Little or no fibrosis, with abundant macroscopic disease (RCRG3); **(J)** Status of five rectal cancer cell lines exposed to irradiation, and survival fraction fitted to the linear quadratic equation; **(K)** Colony formation assays of five rectal cancer cell lines under irradiation interference.

**Figure 2:**
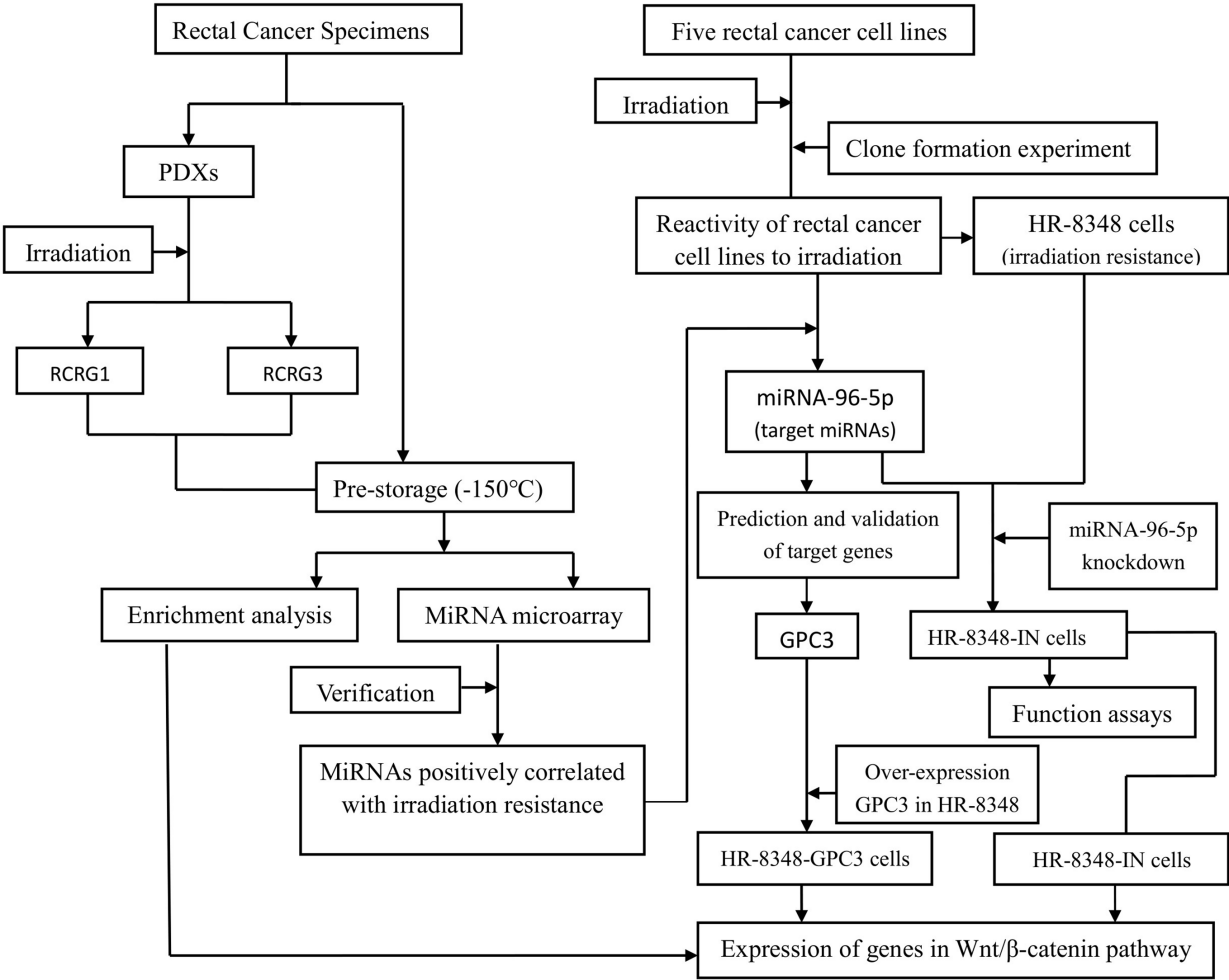
A flowchart of each step in this study.

After the surgical specimen of enrolled patients was isolated, a fresh section of tumor tissue, approximately 10×10×5mm^3^ in size, was immediately cut from the central area of the tumor in a sterile environment (**Figure 1A**). It was then divided into two parts after being washed three times with normal saline. One part was divided into two parts and stored at -150°C refrigerator before being used for miRNA microarray analysis. The other part was cut into 2×2×2mm^3^ segments of tissues and implanted into the dorsal side of the forelimb or hindlimb of BALB/c-nude mice to construct the first generation PDX model. Two-four nude mice were implanted for each specimen, and 1-4 points were implanted for each nude mouse (**Figure 1B**). After the xenograft tumor grew to approximately 10mm in diameter, it was then removed to build a second-generation PDX model on the forelimb shoulder-back of nude mice (**Figure 1C**). Irradiation reactivity experiments were also conducted after the xenograft tumor grew to approximately 10mm in diameter. A total of 259 male SPF grade BALB/c-nude mice, aged 4-6 weeks and weighing 18-20g, were used in this study, all of which were provided by the Institute of Zoology, Chinese Academy of Medical Sciences with all characteristics having been confirmed. Conformity certificate number: SCXK (Beijing) 2015-0013. Animal experiments involved in this study were approved by the Animal Ethics Committee of the Fourth Hospital of Hebei Medical University. Each patient signed an informed consent approved by the Ethics Committee of the Fourth Hospital of Hebei Medical University. In order to clearly illustrate the experimental process, a flow chart of the study design was shown in **Figure 2**. The raw data of key steps in this study can be found in the online link https://pan.baidu.com/s/1-SI1TFD15xCEvdrLLqPfTQ with the extraction code *snls.*

### Evaluation of Irradiation Reactivity of PDX Model

In order to obtain the irradiation response status of PDX models, a single irradiation of 16Gy was administered after the diameter of the implanted tumor reached a diameter 10mm. The scheme is as follows: the limbs of awake nude mice were affixed to foam boards using adhesive tape with tumor surface was covered by a tissue compensation membrane (**Figure 1D**) for radiotherapy with 6MV X-rays. Tumor thickness was defined by the vertical distance ranging from top to bottom. The distance from the irradiation source to the tumor center is 100cm, the dose rate is 500 MU/min, and the size of the irradiation field is 2×2cm. The nude mice were sacrificed 10 days after irradiation, and tissues were excised for HE staining. According to the rectal cancer regression grade (RCRG) standard (23), two experienced pathologists evaluated the irradiation reactivity of the PDX model. Under this scoring criterion, tumor regression was classified into three levels: RCRG 1: Sterilization or only microscopic foci of adenocarcinoma remaining, with marked fibrosis; RCRG 2: Marked fibrosis with macroscopic disease present; RCRG 3: Little or no fibrosis, with abundant macroscopic disease. In this study, we defined RCRG 1 as irradiation sensitive tissue and RCRG 3 as irradiation resistant tissue.

### Screening of Irradiation Resistant Cell Lines

Human rectal cancer cell lines, including HRC-99, HR-8348, SW1463, SW837, and RCM-1, were purchased from Shanghai Fudan University Cell Bank. All cell lines were maintained in our laboratory and cultured in a RMPI 1640 medium (Invitrogen, USA) supplemented with 10% of fetal bovine serum (FBS, Invitrogen, USA), using a humidified 5% CO_2_ incubator at 37°C. Cells were collected during the logarithmic growth phase for subsequent experiments. According to the pre-set irradiation dose gradient, cells were seeded in six-well plates, and the number of cells in a single well of each six-well plate were equal. The irradiation dose corresponding to the number of inoculated cells in each well were as follows: 0Gy 0.15×10^3^ cells, 2Gy 0.3×10^3^ cells, 4Gy 0.6×10^3^ cells, 6Gy 1.2×10^3^ cells, and 8Gy 2.4×10^3^ cells. Cells were incubated for an additional 14 days after irradiation. After incubation, cells were washed with PBS, fixed with paraformaldehyde, and stained with crystal violet solution (0.2%). The survival fraction (SF) was calculated using the numbers of colonies divided by the numbers of cells seeded multiplied by the plating efficiency (PE). Three independent experiments were performed.

### Analysis of miRNA Microarray and Screening of Target Genes

According to the result of RCRG, three pairs of pre-stored tumor tissues corresponding to RCRG 1 and RCRG 3 were taken from -150°C refrigeration. Total RNA was isolated from frozen tumors, five rectal cancer cell lines, and transfected HR-8348 cells, purified using Trizol™ Plus RNA purification Kit (Invitrogen, CA, USA) according to the manufacturer’s recommended protocol, and quantified by UV absorbance at 260 and 280 nm. Denaturing agarose gel electrophoresis was used to evaluate the quality of the samples. Subsequently, miRNAs microarrays were performed by Shanghai Bohao Biotechnology Corporation Co. (http://www.shbiochip.bioon.com.cn *, Shanghai, China*). In this study, 2549 human miRNA precursor loci were annotated using Agilent Human miRNA chip V21.0 database according to the standard protocol (https://www.agilent.com/cs/library/usermanuals/public/G4170-90011.pdf). The detection rate and quality control status of each sample was detailed in **Supplementary Table 1**.

After microarray analysis, quantitative reverse transcription polymerase chain reaction (qRT-PCR) was used for validation of the screening results in tissues and cells, as well as for detection of target genes that may be regulated by miRNA-96-5p. Complementary DNA (cDNA) was synthesized from the total RNA using the GoScript Reverse Transcription System (Promega, Wisconsin, USA) according to the manufacturer’s instruction. TB Green Fast PCR Mix (Takara-Bio-USA) was used as the amplification reagent. The melting curve analysis was performed to confirm the specificity of PCR products. U6 is a highly conserved small nuclear RNA (snRNA), which is relatively stable in different tissues and cells of the same organism, and is one of the most commonly used miRNA housekeeper genes (24, 25). Therefore, in this study, we used U6 as the internal reference of miRNAs. A study on GAPDH mRNA expression in a panel of 72 human tissues by Barber et al. (26) found that there were great differences in the expression of GAPDH between-tissue, but the expression variability of GAPDH gene was generally small within-tissue. Because the tissues and cells involved in this study were all from rectal cancer, we used GAPDH as the internal reference of the target genes expressions of miRNA-96-5p in this study. The total volume of the reaction system was 25μL, including 100 ng/μL cDNA 2μL, 10 μmol/L primer 2μL, Green system 12μL, ddH2O 9μL. Fold change (FC)= ΔCt(RCRG3)/ΔCt(RCRG1) and ΔCt= (Ct miRNA-Ct U6)RCRG3/(Ct miRNA-Ct U6)RCRG1 were used to calculate the expression level multiples of differential miRNAs in irradiation-resistant and irradiation-sensitive cancer tissues. The relative expression levels of miRNAs and mRNAs in cells were calculated by using 2 ^- ΔΔCT^ method. The primers of miRNAs were purchased from RiboBio Biotechnology Co., Ltd. (Guangzhou, China), and the sequences were patented by the company and specific base sequences could not be provided. The mRNAs primers and reaction conditions were listed in **Supplementary Table 2**.

### Functional and Pathway Enrichment Analysis

We analyzed the association of differentially expressed mRNAs with biological processes (BP) and molecular functions (MF) in the Gene Ontology (GO) database (http://geneontology.org). In addition, the Kyoto Encyclopedia of Genes and Genomes (KEGG) pathway analysis of differentially expressed Genes were carried out to comprehensively study the gene and expression information in order to identify the differentially enriched pathways. The enrichment analysis was performed using Fisher’s exact test in cluster Profiler from R/bioconductor (https://www.bioconductor.org). The standard of selection was the number of genes that fall on a GO term/or pathway ≥ 2, with a P-value < 0.05.

### Plasmid Transfection

LV10N-hsa-miRNA-96-5p-inhibitor (5’-AGCAAAAATGTGCTAGTGCCAAA-3’), LV10N-hsa-miRNA-96-5p-NC (5’-TTCTCCGAACGTGTCACGT-3’), LV-SWP-GFP-GPC3 (Forward: 5’-CATCGGTACCATGGCCGGGACCGTGCG-3’, Reverse: 5’-TCGACTCGAGCACCAGGAAGAAGAAGCACACCACCG-3’) and LV-SWP-GFP were purchased from GenePharma Co., Ltd. (Shanghai, China). HR-8348 cells in the logarithmic growth phase were seeded into 24-well plates (0.5×10^5^ cells/well), 0.5mL complete culture medium (10% FBS+RPMI-1640) was added to each well, and incubated at 37°C with 5% CO2 for 24h. Then 10% FBS+DMEM culture medium containing Polybrene (5μg/mL) and the corresponding lentivirus (LV10N-hsa-miRNA-96-5p-inhibitor/-NC, pcDNA3.1-GPC3 vector and pcDNA3.1 empty vector) were used to replace the original medium. After 12-24h, the medium was removed and 0.5mL/well complete medium (10% FBS+RPMI-1640) was added. After 72h, the infection status of the cells was observed under a fluorescence microscope. 0.6µg/ml Purinomycin (Sigma-Aldrich, St. Louis, MO) was added into each well of successfully transfected cells lines. The expression levels of miRNA-96-5p and GPC3 were determined by qRT-PCR analysis.

### Clonogenic Formation Assay of Transfected Cells Under Irradiation Interference

In order to detect the irradiation reactivity of HR-8348 before and after transfection, we performed a clone formation experiment. The experimental procedures were the same as the screening of irradiation resistant cell lines.

### MTS Assays

The proliferation of HR-8348 cells, LV10N-hsa-miR-96-5p-inhibitor HR-8348 (HR-8348-IN) cells, and LV10N-hsa-miR-96-5p-NC HR-8348 (HR-8348-NC) cells were examined by MTS assay (Sigma-Aldrich, USA) at the indicated time points, accordingly to the descriptions of the MTS assays of Cory (27) and McCauley et al. (28). Cells were seeded at a density of 1.0×10^3^ cells/well in 96-well culture plates, and cultured for 0/24/48/72/96h. The absorbance values were determined on a microtiter plate reader (Expire Technology, Perkin Elmer) at 492 nm. Three independent experiments were done in triplicate.

### Wound Healing Assay

HR-8348 cells in different conditions were seeded into 6-well plates at a density of 5.0×10^5/^well and cultured conventionally. When the cells grew to full fusion, a sterile pipette tip was used to lightly scratch the cells at the centre of the 6-well plate. The wounded monolayers were washed with PBS to remove cell debris, and the cells were cultured in an incubator. Closure of the wound was observed under an inverted microscope at 0, 12 and 24h after scratching, and the distance between the two edges was measured. Ten fields of view were randomly selected, and images were acquired at the indicated timepoints. Image−Pro Plus version 5.0 software (Media Cybernetics, Inc., USA) was used to analyze all images.

### Invasion Assay

Cells invasion assays were performed in a 24-well transwell chamber (Corning, USA), containing the 8-μm pore size polycarbonate membrane filter and was precoated with 20μl of Matrigel (Corning, USA) for invasion. Briefly, 1.0×10^5^ cells in different clones were seeded in the upper chambers and incubated in 200 μl RPMI medium (without FBS), while 600 μl medium with 10% FBS was placed in the lower chambers. The plates were incubated for 24h in an incubator. Subsequently, the invaded cells in the lower chamber were fixed with 4% paraformaldehyde for 10 min, stained with 0.1% crystal violet (Beyotime, China) for 5 min and lightly washed with PBS twice. Eventually, the number of invaded cells in five random fields of view were counted and photographed with a fluorescence microscope (Olympus, Japan) at 200×magnification.

### Screening of Target Genes Regulated by miRNA-96-5p

Biological prediction softwares (TargetScan (http://www.targetscan.org), miRDB (http://www.mirdb.org), miRTarbase (http://mirtarbase.mbc.nctu.edu.tw), Tarbase (http://www.microrna.gr/tarbase)) were used to predict the target genes that might be regulated by miRNA-96-5p. Genes that can be predicted by the above four software programs were considered as the preliminary screening results, then the screening results were searched in Pubmed library (https://www.ncbi.nlm.nih.gov/pubmed) to further screen the possible target genes that may be related to biological tumor behavior. The target genes were verified by qRT-PCR and Western blot analysis, and their expression profiles in rectal cancer were obtained by Gene Expression Profiling Interactive Analysis (GEPIA) website (http://gepia.cancer-pku.cn/detail.php).

### Western Blot Analysis

Total proteins from cells were extracted using RIPA lysis buffer containing the protease inhibitor PMSF. Cytoplasmic proteins were extracted using a specialized cytoplasmic protein extraction kit (Sangon Biotech Co., Ltd., Shanghai). Simply, the mixture of cell lysis products and pre-cooled Buffer (1μL DTT, 10μL PMSF, 1μL protease inhibitor, and 5μL phosphatase inhibitor) was centrifuged at 4100 rpm for 15 min at 4°C, and its supernatant was further centrifuged at 18000 RPM for 60min at 4°C again. The final supernatant was cytoplasmic proteins. Western blot was performed routinely, the primary antibodies were as follows: anti-CAV1 (Abcam, ab32577, 1:200), anti-DDIT3 (Abcam, ab11419, 1:1000), anti-PDCD4 (Abcam, ab51495, 1:1000), anti-MBD4 (Abcam, ab227625, 1:200), anti-DAB2 (Abcam, ab33441, 1:500), anti-FOXO3 (Abcam, ab109629, 1:1000), anti-GPC3 (Abcam, ab174851,1:2000), anti-β-catenin (Cell Signal TECHNOLOGY, 8480T, 1:1000), anti-GSK 3β (Cell Signal TECHNOLOGY, 12456T, 1:1000), anti-p-GSK 3β (Cell Signal TECHNOLOGY, 5558T, 1:1000), anti-CD44 (Cell Signal TECHNOLOGY, 37529T, 1:1000), anti-c-Myc (Cell Signal TECHNOLOGY, 5065T, 1:1000) and anti-GAPDH (Bioworld, AP0063, 1:5000). Goat anti-rabbit IgG or goat anti-mouse IgG (1:12,000 dilution; Sigma, USA) were used as the secondary antibody. Bands were visualized using an ECL Western blot detection kit (Amersham, USA). The ECL-based detection was performed with Chemiluminescence Reagent according to the manufacturer’s instructions. The level of GAPDH was used as a loading control.

### Luciferase Reporter Assay

The target gene analysis of miRNA-96-5p was performed using the biological prediction site microRNA.org (http://www.microrna.org/microrna/home.do), and dual-luciferase reporter gene assays were used to determine whether GPC3 was the direct target gene of miRNA-96-5p. The luciferase reporter vectors (pmirGlo-GPC3-3’UTR-WT and pmirGlo-GPC3-3’UTR-MUT) were synthesized by GenePharma Corp. The pRL-TK vector expressing Renilla was used as a reference to control for differences in cell number and transfection efficiency. MiRNA-96-5p mimics and miRNA-NC were co-transfected with luciferase reporter vectors into HR-8348 cells. Then, dual-luciferase reporter assays were performed according to the manufacturer’s instructions (GenePharma Corp. China).

### Statistics

All statistical analyses were carried out using SPSS for Windows version 17.0 (SPSS). Student’s t-test, oneway analysis of variance (ANOVA) and Spearman correlation analysis were used to analyze all of the data. All cell culture experiments were performed in triplicate. Data are presented as mean ± standard deviation (SD). Differences were considered statistically significant for P <0.05.

## Results

### Construction of PDX Models

In our study, a total of 439 sites were implanted in primary transplantation, with a success rate of 58.78% (258/439), and 56 sites were implanted in secondary transplantation, with a success rate of 76.79% (43/56). Finally, PDX models of 29 patients were used in the irradiation experiment. The diameter of the implanted tumors was 10-15mm with a median of 12mm.

### Irradiation of PDX

The volume of xenograft tumor was calculated using the formula for a spheroid “volume = length × height × width × π/6” prior to and 10 days after irradiation. As shown in **Table 1**, there was no significant difference between the volumes of implanted tumors before and after radiation *in vivo*. After the measurement of gross volume, the nude mice were sacrificed and the tumor tissues were stripped. All specimens were found to have different degrees of liquefaction and degeneration. Among them, 3 specimens were mostly replaced by liquefied and necrotic tissues (**Figure 1E**), and the remaining specimens were tumors. After cleaning the liquefied necrotic tissue on tumor surface, the volume of the isolated tumor tissue was measured (**Figure 1F**) and compared with the volume of the tumor before and after irradiation, and there was a large difference between them (P<0.001) (**Table 1**). The response of the implanted tumor to irradiation was evaluated according to the RCRG standard. It was found that 17.24% (5/29) of specimens were RCRG 1, 72.41% (21/29) specimens were RCRG 2, and 10.34% (3/29) specimens were RCRG 3 (**Figures 1G–I**).

**Table 1:** Volume comparison of implanted tumors before and after irradiation.

Groups	$\bar{\text{X}}$ ± SD(mm^3^)	95% CI	F	*P* value
Volume *in vivo* before irradiation	696.33 ± 315.32	599.28-793.37	0.39*	0.53*
Volume *in vivo* after irradiation	651.93 ± 338.89	547.63-756.23	49.54#	0.00#
Volume *in vitro* after irradiation	194.19 ± 258.88	114.52-273.86	65.14^	0.00^

, Mean value; SD, Standard deviation; CI, confidence interval; F, the statistic of one way ANOVA; *Tumor volume before irradiation in vivo *vs*. tumor volume in vivo after irradiation; #Tumor volume in vivo before irradiation *vs*. tumor volume *in vitro* after irradiation; ^Tumor volume *in vivo* after irradiation vs. tumor volume *in vitro* after irradiation.

### Irradiation Response of Rectal Cancer Cells

The colony-forming ability and survival fraction of rectal cancer cell lines were shown in **Table 2** and **Figures 1J, K**. HR-8348 cells presented with the highest resistance to irradiation than other cell lines (P<0.05). The trend of radio-resistant in these five cell lines was HR-8348> HRC-99> RCM-1> SW837> SW1463.

**Table 2:** Related parameters of cell survival curve standard model.

Dose (Gy)	HR-8348	HRC-99	RCM-1	SW837	SW1463	*P* value
D0	1.839 ± 0.108	2.237 ± 0.181	2.403 ± 0.291	2.789 ± 0.243	2.621 ± 0.109	0.002
Dq	4.091 ± 0.110	3.443 ± 0.281	2.847 ± 0.330	1.473 ± 0.409	0.463 ± 0.279	0.000
D37	5.930 ± 0.013	5.679 ± 0.106	5.250 ± 0.079	4.262 ± 0.167	3.084 ± 0.375	0.000
SF2	0.919 ± 0.026	0.852 ± 0.032	0.788 ± 0.047	0.635 ± 0.038	0.506 ± 0.035	0.000

D0, reciprocal of dose slope; Dq, quasithreshold dose; D37, irradiation dose at 37% survival fraction; SF2, survival fraction at 2 Gy.

### Differential miRNA Expression Profiles Between RCRG 1 and RCRG 3

**Figure 3:**
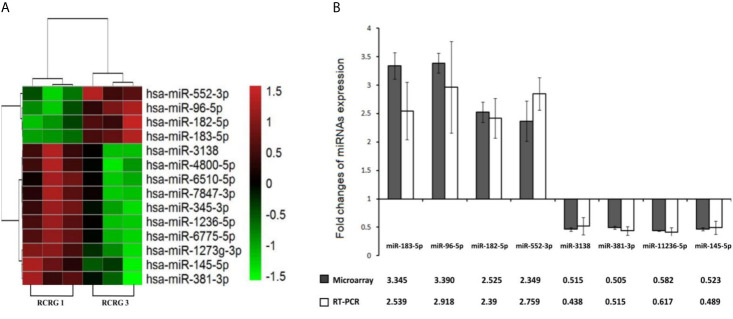
Differential miRNAs expression between RCRG1 and RCRG3. **(A)** MiRNAs microarray was used to detect the differential expression of miRNAs between RCRG1 and RCRG3, the scale bar indicates miRNAs expression level: red represented high expression, green represented low expression; **(B)** Differential expression miRNAs were confirmed by qRT-PCR.

The miRNAs microarrays indicated that a total of 14 miRNAs were differentially expressed with a fold change value of ≥1.5, 4 up-regulated and 10 down-regulated (**Figure 3A**). Furthermore, using qRT-PCR, we confirmed that the relative expression levels of 4 up-regulated and 4 down-regulated miRNAs were consistent with the microarray data (**Figure 3B**).

### miRNAs Expression in Rectal Cancer Cell Lines

**Figure 4:**
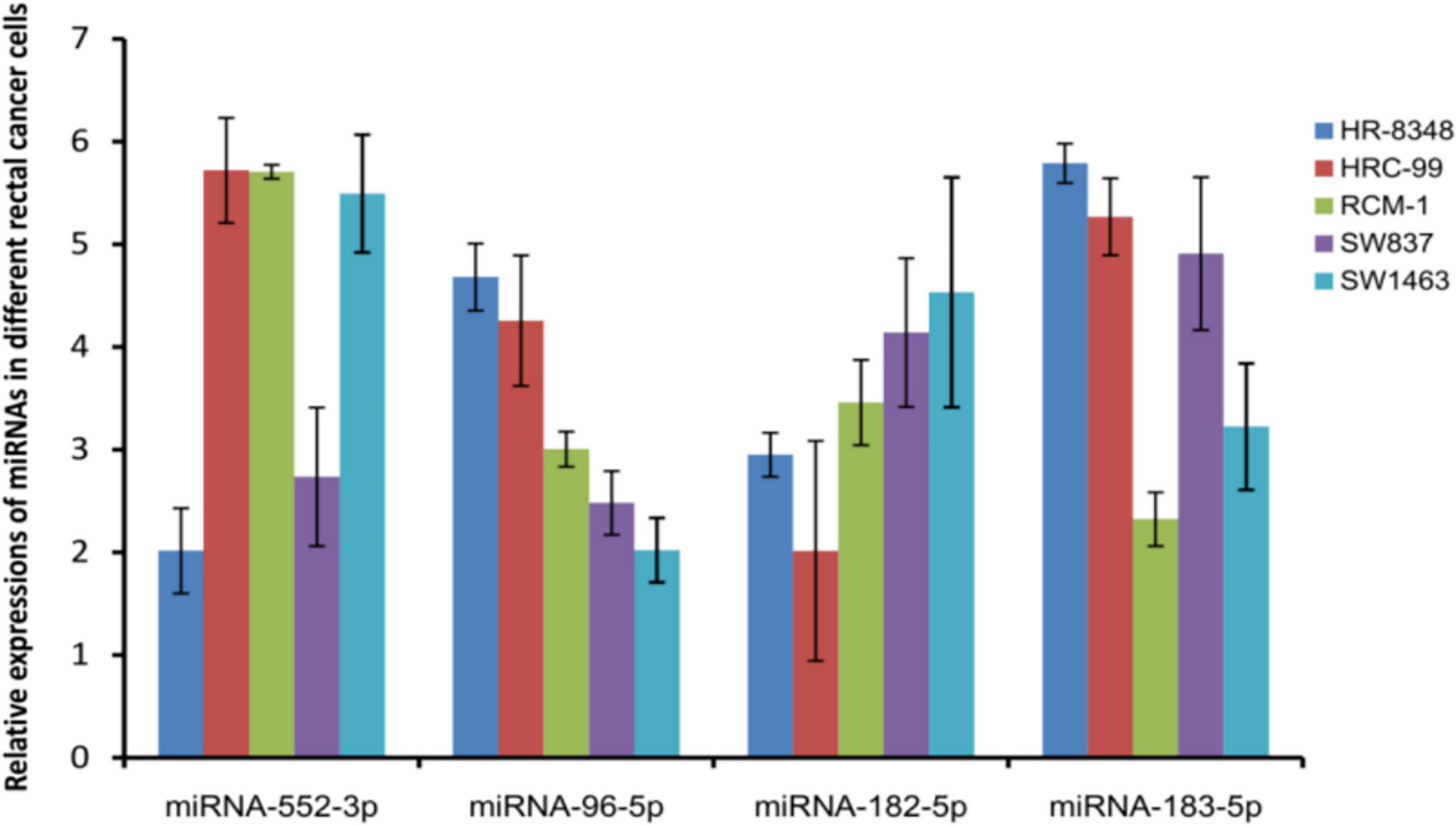
Detecting the expression of miRNAs positively correlated with irradiation resistance in rectal cancer cell lines showed that the expression of miRNA-96-5p was positively correlated with the radiation resistance of the cells.

miRNAs positively related to radio-resistance were tested in each cell lines using the qRT-PCR method. Interestingly, among these four miRNAs, the expression level of miRNA-96-5p was not only positively correlated with the radio-resistance, but also consistent with the trend of the irradiation resistance of these five cell lines (**Figure 4**). Compared to the most radio-sensitive SW1463 cells, the radio-resistant HR-8348 cells expressed approximately 2.3 fold of miRNA-96-5p (**Table 3**). The Spearman correlation analysis verified a positive correlation with irradiation resistance and expression of miRNA-96-5p in rectal cancer cells (r_s_=0.938, P =0.000).

**Table 3:** Expression of miRNAs positively correlated with irradiation resistance in different cell lines.

miRNAs	SW1463	SW837	RCM-1	HRC-99	HR-8348	r_s_	*P* value
miRNA-552-3p	5.4 ± 0.57	2.7 ± 0.67	5.7 ± 0.07	5.7 ± 0.51	2.0 ± 0.41	-0.262	0.346
miRNA-96-5p	2.02 ± 0.31	2.48 ± 0.31	3.00 ± 0.17	4.26 ± 0.64	4.67 ± 0.33	0.938	0.00
miRNA-182-5p	4.5 ± 1.12	4.1 ± 0.72	3.4 ± 0.41	2.0 ± 1.07	2.9 ± 0.21	-0.786	0.001
miRNA-183-5p	3.2 ± 0.61	4.9 ± 0.74	2.3 ± 0.26	5.2 ± 0.37	5.7 ± 0.19	0.578	0.024

r_s_, Spearman rank correlation coefficient.

### Inhibition of miRNA-96-5p Decreases Irradiation Resistance and Promotes Nonaggressive Phenotype in HR-8348 Cells

**Figure 5:**
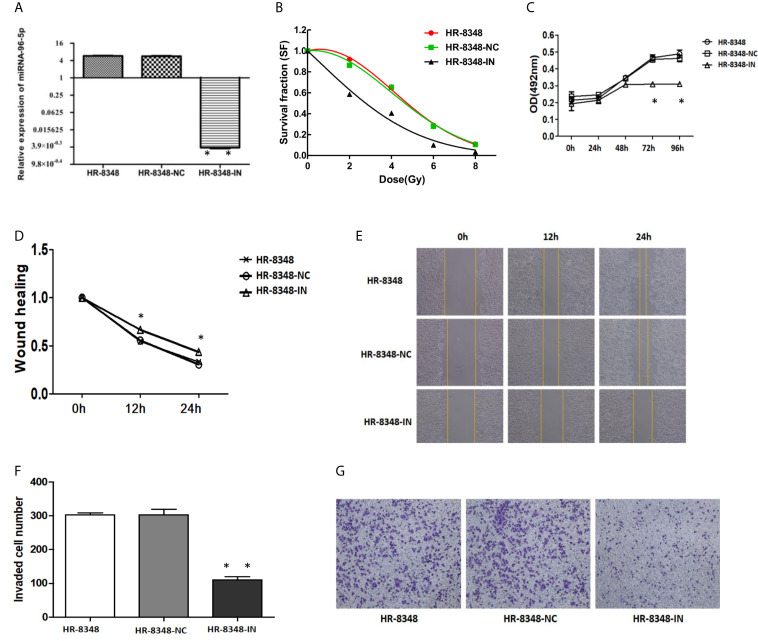
Changes in irradiation reactivity and biological behavior of HR-8348 cells before and after miRNA-96-5p knockdown. **(A)** MiRNA-96-5p was significantly down-regulated in HR-8348-IN cells; **(B)** MiRNA-96-5p low-expression in radio-resistant HR-8348 cells results in increased sensitivity to irradiation, and survival fraction fitted to the linear quadratic equation; **(C)** The effect of miRNA-96-5p on rectal cancer cells growth, as measured using the MTS assay; **(D–G)** The role of miRNA-96-5p in migration and invasion of rectal cancer cells was detected by wound healing test and Transwell assays *P < 0.05, **P < 0.01.

Next, we examined the potential role of miRNA-96-5p by suppressing miRNA-96-5p expression in HR-8348 cells. The expression of miRNA-96-5p was successfully down-regulated in its inhibitor transfected HR-8348 (HR-8348-IN) cells (5.75± 0.45 *vs*. 0.0036 ± 0.00095) (**Figure 5A**). Through the clone formation experiment under irradiation, it was found that the irradiation resistance of HR-8348 cells was decreased after inhibiting the expression of miRNA-96-5p (**Figure 5B**). In addition, we also conducted a series of cell function experiments to explore the role of miRNA-96-5p. MTS assays showed that the down-regulation of miRNA-96-5p significantly reduced the proliferation rate of HR-8348 cells (**Figure 5C**). Through wound healing assay and invasion assays, we found that the down-regulation of miRNA-96-5p significantly reduced the migration and invasion ability of HR-8348 cells (**Figures 5D–G**), suggesting that miRNA-96-5p has a positive effect on the aggressiveness of rectal cancer cells.

### miRNA-96-5p Targets GPC3 in HR-8348 Cells

**Figure 6:**
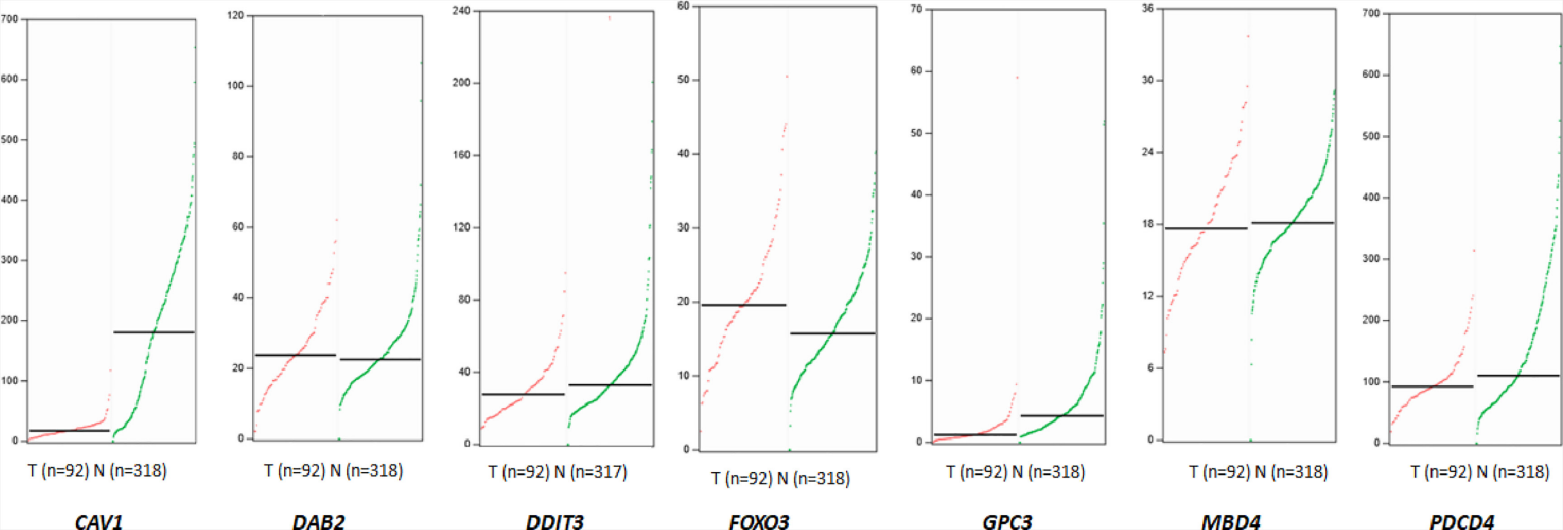
The expression profiles of seven target genes that may be regulated by miRNA-96-5p obtained from the GEPIA website in rectal cancer tissues and normal tissues. The median values of CAV1, DAB2, DDIT3, FOXO3, GPC3, MBD4 and PDCD4 in rectal cancer tissues and normal tissues were 17.16 and 180.87, 23.78 and 22.54, 27.78 and 33.00, 19.58 and 15.76, 1.24 and 4.3, 17.76 and 18.13, 92.51 vs.110.35, respectively. The red line represents the tumor, and the green line represents normal tissue.

**Figure 7:**
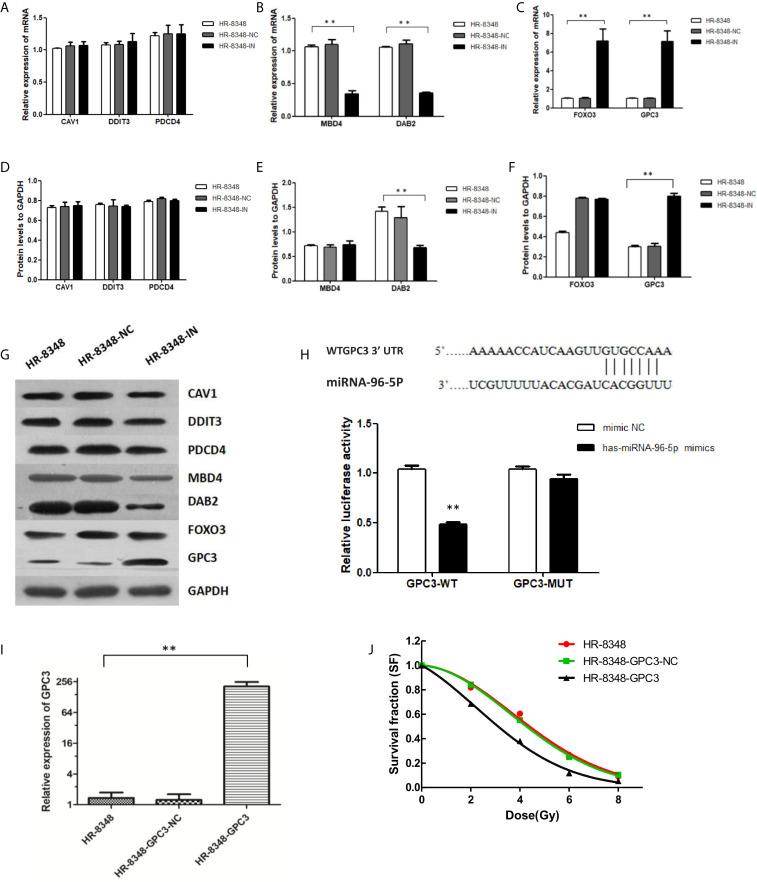
Screening of target genes regulated by miRNA-96-5p and the effect of up-regulation of GPC3 expression on radiation reactivity of HR-8348 cells. **(A–C)** qRT-PCR assays were used to detect the expression levels of target genes in rectal cancer cells before and after miRNA-96-5p knockdown, and the mRNA expressions of FOXO3 and GPC3 were significantly up-regulated in HR-8348-IN cells; **(D–G)** Western blot assays were used to detect the expression of target genes in rectal cancer cells before and after miRNA-96-5p knockdown, and the protein expression of GPC3 was not significantly changed in HR-8348-NC cells, but was significantly up-regulated in HR-8348-IN cells; **(H)** Luciferase activity of the WT and mutant GPC3 3′UTR co-transfected with miRNA-NC and miRNA-96-5p mimics, the GPC3-WT group was inhibited, but not in the GPC3-MUT group; **(I)** GPC3 was significantly up-regulated in HR-8348-GPC3 cells; **(J)** GPC3 over-expression in HR-8348 cells results in increased sensitivity to irradiation, and survival fraction fitted to the linear quadratic equation. **P < 0.01.

A total of 3419 genes that might be regulated by miRNA-96-5p were predicted using four different bioinformatics software programs. CAV1, DAB2, DDIT3, EDEM1, FoxO3, GPC3, MBD4, PDCD4, SLC25A25 and ZEB110 genes were all cross-predicted in these prediction tools. We further focused on seven genes including CAV1, DAB2, DDIT3, FOXO3, GPC3, MBD4, and PDCD4, being that they have been reported to play important roles in tumorigenesis. The expression profiles of the above seven genes obtained from the GEPIA website showed that GPC3 had the lowest expression in rectal cancer (**Figure 6**). Moreover, the target gene validation results showed that only GPC3 was up-regulated in HR-8348-IN cells (**Figures 7A–G**). These results suggest that GPC3 may be the target gene regulated by miRNA-96-5p in HR-8348 cells. To determine whether GPC3 gene is directly regulated by miRNA-96-5p, we examined the effect of miRNA-96-5p on the activity of luciferase driven by GPC3 3’-UTR. The results showed that luciferase activity was significantly inhibited in the GPC3-WT group but has no effect in the GPC3-MUT group (**Figure 7H**). These findings implied that GPC3 might be a direct target gene of miRNA-96-5p.

### Over-Expression of GPC3 Enhanced the Radio-Sensitivity of HR-8348 Cells

HR-8348 cell lines with GPC3 stable over-expression (HR-8348-GPC3) were obtained by lentivirus transfection, and the transfection efficiency was 1.36 ± 0.38 *vs*. 207.01 ± 46.19 (**Figure 7I**). Furthermore, we found that HR-8348-GPC3 had significantly higher radiation sensitivity than HR-8348 cells (**Figure 7J**).

### Down-Regulation of miRNA-96-5p Inhibited the Activity of the Wnt/β-Catenin Signal Transduction Pathway

**Figure 8:**
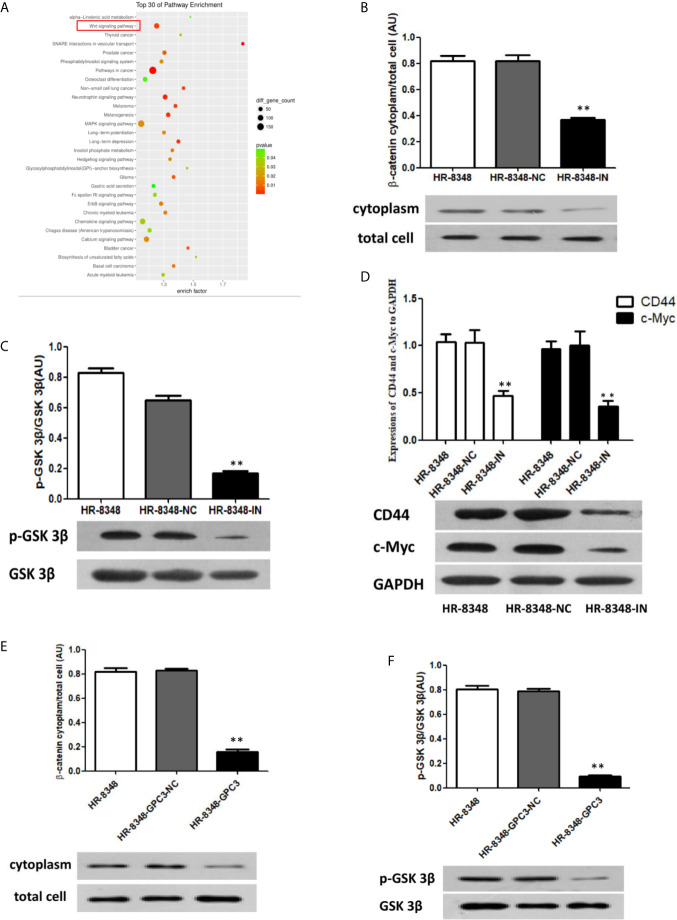
Effect of miRNA-96-5p on the status of Wnt/β-catenin signal transduction pathway in rectal cancer cells. **(A)** Screening of enriched signal pathway indicated that Wnt signal pathway was one of the important factors affecting irradiation resistance of rectal cancer. **(B, C)** Down-regulation of miRNA-96-5p significantly reduced the expression of β-catenin in the cytoplasm and the phosphorylation level of GSK3β in rectal cancer cells. **(D)** After the down-regulation of miRNA-96-5p, the expression of CD44 and c-Myc was significantly reduced in rectal cancer cells. **(E, F)** GPC3 over-expression significantly reduced the expression of β-catenin in the cytoplasm and the phosphorylation level of GSK3β in rectal cancer cells. **P < 0.01.

From the results of an enriched signal transduction pathways analysis in rectal cancer tissues with different irradiation resistance abilities, we found that Wnt was one of the most significant signal transduction pathways (**Figure 8A**). Therefore, the expression of key genes and downstream genes of canonical Wnt signal pathway were detected by Western blot analysis. In our results, the relative expression levels of β-catenin, a key gene in Wnt signaling pathway, in HR-8348 cells before and after transfection (HR-8348, HR-8348-NC and HR-8348-IN) were 0.804 ± 0.035, 0.767 ± 0.253 and 0.781 ± 0.0185, respectively, without statistical difference (P>0.05). Since it is known from previous reports (29) that the expression and accumulation level of β-catenin in the cytoplasm is an important factor affecting the activity of the Wnt/β-catenin signal transduction pathway, we further checked it in the cytoplasm of the three groups of cells, and found that the levels were 0.631 ± 0.05, 0.665 ± 0.038, and 0.321 ± 0.0108, respectively, and the ratios of β-catenin expression in the cytoplasm to total cell were 0.81 ± 0.0442, 0.823 ± 0.0321 and 0.392 ± 0.0088, respectively, (P<0.05) (**Figure 8B**). These results suggested that β-catenin expression in the cytoplasm of HR-8348 cells was inhibited with the down-regulated expression of miRNA-95-5p.

To investigate the mechanisms of the down-regulated β-catenin in the cytoplasm of HR-8348-IN cells, we detected the expression of GSK 3β and its phosphorylation, key regulatory factors of β-catenin. The relative expression levels of GSK 3β in the three cell lines were 0.979 ± 0.007, 1.06 ± 0.014, and 0.847 ± 0.044, respectively (P>0.05). However, the expression level of p-GSK 3β in HR-8348-IN cells was significantly lower than that in the other two groups (P<0.05), and the expression levels in each group were 0.872 ± 0.056, 0.698 ± 0.073, and 0.143 ± 0.003, respectively. The ratios of p-GSK 3β to GSK 3β in three groups were 0.82 ± 0.0314, 0.668 ± 0.0216 and 0.173 ± 0.0194, respectively (P<0.05) (**Figure 8C**). These results suggested that the phosphorylation level of GSK 3β was decreased in HR-8348 cells under the down-regulation of miRNA-96-5p expression.

Based on the above results, we speculated that the down-regulation of miRNA-96-5p in HR-8348 cells might inhibit the activity of the Wnt/β-catenin signaling pathway. To verify this hypothesis, we examined the expression of CD44 and c-Myc, the downstream genes of Wnt/β-catenin pathway. Our results showed that the expressions of these two genes were significantly decreased in HR-8348-IN cells, the expression levels of CD44 in the three cell lines were 1.016 ± 0.053, 1.045 ± 0.163, 0.475 ± 0.069 and c-Myc were 0.940 ± 0.069, 1.004 ± 0.180, 0.343 ± 0.052, respectively (**Figure 8D**).

### Effect of Up-Regulation of GPC3 to Wnt/β-Catenin Signal Transduction Pathway

To determine whether GPC3 was involved in the change of Wnt/β-catenin activity, the expression of β-catenin and GSK 3β in HR-8348 before and after GPC3 up-regulated were examined. We found that the ratios of β-catenin expression in cytoplasm to total cells were 0.818 ± 0.0275, 0.827 ± 0.0153, and 0.159 ± 0.0176 (P<0.05) (**Figure 8E**), and p-GSK 3β to GSK 3β was 0.802 ± 0.0301, 0.79 ± 0.017, and 0.093 ± 0.0121, respectively (P<0.05) (**Figure 8F**). These results indicated that the activity of the Wnt/β-catenin signal transduction pathway in HR-8348 cells was inhibited under the up-regulation of GPC3 expression.

## Discussion

For patients with locally advanced rectal cancer, nCRT combined with TME is the most conventional treatment according to current guidelines and clinical practice. However, not everyone can benefit from nCRT. Therefore, it is of great clinical significance to determine an efficient method or effective indicators that can be used to identify patients who are resistant to radiotherapy prior to initial treatment. In this study, we took samples from the central area of newly excised tumor specimens. By using this method, the difference in biological behavior between the pre-storage specimens and the xenograft tumor will be reduced due to spatial heterogeneity.

According to the screening results of PDX in response to irradiation, microRNA differential screening was performed on the pre-storage samples corresponding to the irradiation resistance and sensitivity models. Our results showed that the expression of miRNA-552-3p, miRNA-96-5p, miRNA-182-5p and miRNA-183-5p were significantly up-regulated in irradiation resistant tissues. Interestingly, in the results of Li et al.’s screening of differential miRNAs between rectal cancer tissues and normal rectal tissues, these four miRNAs also showed up-regulation in cancer tissues (30). In fact, previous studies on the irradiation resistance of rectal cancer to miRNAs resulted in an inconsistency of outcomes reached by different researchers. For example, in the studies of Drebber et al. (31), Della Vittoria Scarpati et al. (32), and Kheirelseid et al. (33), it was not found that the same miRNAs were repeatedly screened, and even in two similar screening studies of the same research group, no identical miRNAs were found (34, 35). Some researchers believe that the reason for this phenomenon may be related to the quality and preservation of the specimen’s condition (36), in addition, the difference between the number of samples submitted for inspection and the actual inspection platform may also be an important influencing factor.

After validation of the four miRNAs, we found that the expression of miRNA-96-5p was positively correlated with the radiation resistance of rectal cancer cells. MiRNA-96-5p is a member of the miRNA-183 family, which are located in a cluster on human chromosome 7q32. Studies have found that high expression of miRNA-96 in gastric cancer (37), lung cancer (38), colon cancer (39), ovarian cancer (40) and other malignant tumors are closely related to the proliferation, invasion and migration of cancer cells. In terms of response to irradiation, Vahabi et al. (41) found that the over-expression of miRNA-96-5p in head and neck squamous carcinoma cells was involved in the regulation of adhesion, extracellular matrix and PI3K-Akt signaling pathway, which enhanced the migration ability of carcinoma cells as well as their resistance to radiotherapy and chemotherapy. So far, however, there is no report on the relationship between miRNA-96 and irradiation resistance of rectal cancer. In this study, we found that HR-8348 cells had high irradiation resistance by comparing the response ability of different rectal cancer cell lines to irradiation. Among the four miRNAs positively correlated with irradiation resistance, only the expression level of miRNA-96-5p was consistent with the trend of irradiation resistance of five rectal cancer cell lines. Therefore, miRNA-96-5p was identified as the research object of the cell experiment in this study.

In our study, LV10N-has-miR-96-5p-inhibitor lentivirus plasmid was constructed, and HR-8348 cell lines with low-expression of miRNA-96-5p were obtained. The clone formation experiment under irradiation interference and cell function experiments further confirmed that the high expression of miRNA-96-5p is one of the reasons that lead to the high irradiation resistance and aggressive phenotype of HR-8348 cells. Therefore, we believe that the up-regulated expression of miRNA-96-5p in rectal cancer cells plays a role of oncogene, and the intervention of its expression might become a new way to increase the irradiation sensitivity of rectal cancer cells.

To our knowledge, the regulation of biological cell functions by miRNA is realized through the expression changes of target genes. In this study, we screened and verified the target genes regulated by miRNA-96-5p, and found that GPC3 was the target gene directly regulated by miRNA-96-5p in HR-8348 cells. After the over-expression of GPC3 gene in HR-8348 cells, we found that the radiation sensitivity of rectal cancer cells was significantly increased, which was consistent with our results of miRNA-96-5p knockdown. It further proved that the regulation of GPC3 by miRNA-96-5p in HR-8348 cells was one of the reasons for its irradiation resistance. Current studies show that the expression level and the biological role of GPC3 in different cancer species are quite different, for example, it was highly expressed in Wilms tumor (42), yolk sac tumor (43), hepatocellular carcinoma (44) and clear cell ovarian cancer (45); while its expression in mesothelioma (46), lung adenocarcinoma (47), clear cell renal cancer (48), gastric cancer (49), and breast cancer (50) was inhibited. In hepatocellular carcinoma, the up-regulated expression of GPC3 was closely associated with malignant behavior and poor prognosis of tumors (51, 52); while in breast cancer, its over-expression not only inhibits tumor invasion and metastasis, but also was related to the decrease of cell viability and survivability, the increased homogeneous adhesion (50) along with the transformation of mesenchymal cells into epithelial cells (53). At present, there are not many reports regarding the relationship between GPC3 and the biological behavior of colorectal cancer. Yuan et al. found that the increased expression of GPC3 in colorectal cancer was related to tumor invasion and lymph node metastasis (54), while Foda et al. believed that GPC3 and E-cadherin expression in colonic non-mucinous adenocarcinoma were significantly correlated, but not related to DFS and OS (55). Therefore, the relationship between the expression of GPC3 and the biological behavior of colorectal cancer remains to be further studied. In our study, we found that HR-8348 cells with down-regulated miRNA-96-5p was significantly reduced in their proliferation, migration, and invasion abilities compared with the untransfected HR-8348 cells, while GPC3 expression was significantly up-regulated in the transfected cells, which indirectly suggested that GPC3 might play a nonaggressive phenotype effect in rectal cancer cells.

Activation of the Wnt/β-catenin signal transduction pathway promotes irradiation resistance in a variety of malignant tumors, including rectal cancer, which has been demonstrated in several studies (56–59). The present study also supports this view by analyzing the enrichment signaling pathways that influence irradiation resistance in rectal cancer. Our results showed that neither the expression of β-catenin nor GSK-3β found statistical differences among the three cell lines. However, further detection of β-catenin expression in the cytoplasm found that the ratio of β-catenin expression in the cytoplasmic to the total expression in HR-8348-IN cells was 2 times lower than that in HR-8348 cells, and the ratio of p-GSK-3β to GSK-3β in HR-8348-IN cells was nearly 5 times lower than in HR-8348 cells. Although no studies have been reported on the regulation of miRNA-96 on β-catenin, GSK-3β and p-GSK-3β, However, based on the above results, it can be speculated that miRNA-96-5p may affect the expression of key genes on the Wnt/β-catenin signal transduction pathway through some direct or indirect way.

To confirm this hypothesis, we examined the expression of c-Myc (60, 61) and CD44 (62–64), downstream genes of the Wnt/β-catenin signaling pathway, and found that the expression levels of both genes in HR-8348-IN cells were significantly lower than that in HR-8348 cells. At present, the existence of tumor stem cells in solid tumors is an important factor leading to irradiation resistance of patients has become the basic consensus in the radiotherapy field. Previous studies have shown that both c-Myc (65, 66) and CD44 (67–71) have the characteristics of tumor stem cell marker factors, so we believe that the two genes are eligible for irradiation resistance markers. In fact, the positive correlation between the expression level of these two genes and tumor irradiation resistance has also been confirmed in several studies (72–77). Therefore, we speculated that the regulation of miRNA-96-5p on irradiation resistance of rectal cancer cells was not only related to the abnormal activation of Wnt signal transduction pathway, but also may be related to the “stem” characteristics of tumor cells.

In this study, we found that GPC3 was the target gene directly regulated by miRNA-96-5p in HR-8348 cells. Up to now, there has been no report on the relationship between GPC3 and tumor response to irradiation. Previous studies have shown that the expression of GPC3 was indeed correlated with the activity of the Wnt/β-catenin signaling pathway (78–82). However, studies found that the relationship between GPC3 and Wnt/β-catenin in malignant tumors varies according to tumor types. For example, Gao et al. found that GPC3 can regulate tumor proliferation and progression through activation of Wnt/β-catenin signaling pathway in liver cancer (83). Wang et al. found that the up-regulation of GPC3 in lung squamous cell carcinoma enhanced the expression of β-catenin, promoted cell growth and tumorigenesis, and inhibited cell apoptosis (84). In contrast to these results, Stigliano et al. found that GPC3 inhibited the Wnt/β-catenin signaling pathway involved in the regulation of breast cancer cell proliferation and survival (85). As previously mentioned, a large number of studies have confirmed that there is a positive correlation between Wnt/β-catenin activation and tumor irradiation resistance. Our results showed that the up-regulated of GPC3 inhibited the expression of key genes on the Wnt/β-catenin pathway and enhanced the radiation sensitivity of HR-8348, which was consistent with the results after the down-regulation of miRNA-96-5p. Therefore, we speculated that there may be a regulatory system of miRNA-96-5P/GPC3/Wnt/β-catenin in rectal cancer cells. This system may be an important factor in regulating the radiation responsiveness ability of rectal cancer cells.

Although we obtained miRNAs that are positively correlated with radiation resistance in rectal cancer, there are still some limitations in this study. First of all, due to the relatively small sample size used for miRNAs microarray screening, the generality of this result still needs to be verified in subsequent studies. In addition, among the miRNAs which were positively correlated with radiation resistance screened in this study, only miRNA-96-5p was successfully verified in cell experiments. Previous studies (86–88) have shown that miRNA-96-5p, as a member of the miRNA-183 family, is often co-expressed with miRNA-182 and miRNA-183 in the same tumor. Although this phenomenon was also confirmed in our miRNAs microarray results, the co-expression phenomenon was not detected in subsequent cell experiments, the mechanism needs to be further explored. Thirdly, differentially expressed miRNA-96-5p was found to affect the expression of β-catenin in the cytoplasm and the phosphorylation level of GSK 3β, however, the main purpose of this study was to screen out the miRNAs which were positively correlated with irradiation resistance of rectal cancer and the target genes regulated by them, the regulatory effect (direct or indirect) of miRNA-96-5p on these genes were not further analyzed and need to be explored in follow-up studies.

In conclusion, the present study showed that miRNA-552 and miRNA-183 families play a positive regulatory role in irradiation resistance of rectal cancer, and abnormal activation of Wnt/β-catenin signal transduction pathway is involved in this process. The down-regulated expression of GPC3 gene directly regulated by miRNA-96-5p might be one of the reasons for irradiation resistance of rectal cancer cells, and this effect may be related to the activity changes of Wnt/β-catenin signal transduction pathway. MiRNAs, which are related to irradiation resistance of rectal cancer in this study, may serve as a reminder for this field. But the results of the regulation of miRNA-96-5p on the GPC3 gene need to be verified in clinical practice.

## Data Availability Statement

The datasets presented in this study can be found in online repositories. The names of the repository/repositories and accession number(s) can be found in the article/**Supplementary Material**.

## Ethics Statement

The studies involving human participants were reviewed and approved by the ethics committee of the Fourth Hospital of Hebei Medical University. The patients/participants provided their written informed consent to participate in this study. The animal study was reviewed and approved by the ethics committee of the Fourth Hospital of Hebei Medical University.

## Author Contributions

GW was fully responsible for the design and implementation of the project. FW was responsible for the implementation of the project and the writing of the paper. BW and XZ were responsible for the implementation and literature retrieval of cell experiments. CY was in charge of data statistics and analysis. SR was in charge of animal experiments. CZ was in charge of specimen acquisition. JW was responsible for the radiation of cells and PDX models. YY is in charge of cell culture. All authors contributed to the article and approved the submitted version.

## Conflict of Interest

The authors declare that the research was conducted in the absence of any commercial or financial relationships that could be construed as a potential conflict of interest.

## Publisher’s Note

All claims expressed in this article are solely those of the authors and do not necessarily represent those of their affiliated organizations, or those of the publisher, the editors and the reviewers. Any product that may be evaluated in this article, or claim that may be made by its manufacturer, is not guaranteed or endorsed by the publisher.
