# Supplementary material for: Screening of MicroRNA Related to Irradiation Response and the Regulation Mechanism of miRNA-96-5p in Rectal Cancer Cells
Source: Front Oncol. 2021 Aug 11;11:699475. doi: 10.3389/fonc.2021.699475 (PMC8386172; doi:10.3389/fonc.2021.699475)
Supplement: Supplementary file 1 [file Table_1.docx]

**Supplementary Table 1. Quality control status of RNA and miRNA microarrays**

| Raw data file name | A_260_/ A_280_ | RIN | 28S/18S | CV (%) | Detection rate (%) |
| --- | --- | --- | --- | --- | --- |
| 257015612965_S01_miRNA_107_Sep09_105_1_1 | 2.14 | 8.3 | 1.9 | 6.57 | 30.8 |
| 257015612966_S01_miRNA_107_Sep09_105_2_1 | 2.15 | 9.0 | 1.8 | 7.82 | 21.77 |
| 257015612966_S01_miRNA_107_Sep09_105_2_2 | 2.15 | 8.9 | 1.8 | 6.58 | 19.54 |
| 257015612965_S01_miRNA_107_Sep09_105_2_3 | 2.13 | 9.0 | 1.8 | 6.47 | 25.58 |
| 257015612966_S01_miRNA_107_Sep09_105_1_2 | 2.14 | 8.6 | 1.9 | 6.49 | 25.26 |
| 257015612965_S01_miRNA_107_Sep09_105_1_3 | 2.14 | 8.9 | 1.9 | 6.13 | 27.27 |

**Note:** A260/A280 is the ratio of the absorption value of RNA at 260nm to that at 280nm. This value is used to indicate the purity of RNA. The A260/A280 value of pure RNA is about 2.0. RIN is an abbreviation of RNA Integrity Number, and it is an indicator of RNA integrity. The value ranges from 0 to 10, and the higher the number, the better the integrity of RNA. 28S/18S is an indicator to measure RNA integrity. If the value of 28S/18S is between 1.8 and 2.0, it indicates that RNA integrity is good and basically no degradation occurs. CV is the abbreviation of coefficient of variation, and it is the index to judge the stability of the detection system. CV <10% indicates that the stability of the microarray and technology are good.
