# Supplementary material for: Screening of MicroRNA Related to Irradiation Response and the Regulation Mechanism of miRNA-96-5p in Rectal Cancer Cells
Source: Front Oncol. 2021 Aug 11;11:699475. doi: 10.3389/fonc.2021.699475 (PMC8386172; doi:10.3389/fonc.2021.699475)
Supplement: Supplementary file 2 [file Table_2.docx]

**Supplementary Table 2. The primer sequences of miRNA and mRNA primer sequences and the qRT-PCR reaction conditions used in this study**

| Gene | Primers | Annealing temperature  (℃) |
| --- | --- | --- |
| miRNAs | The sequences were patented by the RiboBio Biotechnology Co. | 55 |
| PDCD4 | R: 5’-AAACCCTGCAGAAAATGCTGG-3’ | 60 |
|  | F: 5’- TGCCAACACTGGTACTCCAC-3’ |  |
| FOXO3 | R: 5’-TCACGCACCAATTCTAACGC-3’ | 60 |
|  | F: 5’- CACGGCTTGCTTACTGAAGG-3’ |  |
| CAV1 | R: 5’-CATCCCGATGGCACTCATCTG-3’ | 60 |
|  | F: 5’- TGCACTGAATCTCAATCAATCAGGAAG-3’ |  |
| GPC3 | R: 5’-GTGCTTTGCCTGGCTACATC-3’ | 60 |
|  | F: 5’- TCCACGAGTTCTTGTCCATTC-3’ |  |
| DAB2 | R: 5’-GTAGAAACAAGTGCAACCAATGG-3’ | 60 |
|  | F: 5’- GCCTTTGAACCTTGCTAAGAGA-3’ |  |
| DDIT3 | R: 5’-GAACGGCTCAAGCAGGAAATC-3’ | 60 |
|  | F: 5’- TTCACCATTCGGTCAATCAGAG-3’ |  |
| MBD4 | R: 5’-TCTAGTGAGCGCCTAGTCCCAG-3’ | 60 |
|  | F: 5’- TTCCAATTCCATAGCAACATCTTCT-3’ |  |
| GAPDH | R: 5’-AGGTGAAGGTCGGAGTCAACG-3’ | 60 |
|  | F: 5’- AGGGGTCATTGATGGCAACA-3’ |  |
